# Clinical and Radiographic Evaluation of Short- and Long-Term Outcomes of Different Treatments Adopted for Elbow Medial Compartment Disease in Dogs

**DOI:** 10.3390/vetsci9020070

**Published:** 2022-02-07

**Authors:** Daniele Serrani, Sara Sassaroli, Francesco Gallorini, Alberto Salvaggio, Adolfo Maria Tambella, Ilaria Biagioli, Angela Palumbo Piccionello

**Affiliations:** 1 Southern Counties Veterinary Specialists, Forest Corner Farm, Hangersley, Ringwood, Hampshire BH24 3JW, UK; 2 Scuola di Bioscienze e Medicina Veterinaria, Università Degli Studi di Camerino, 62024 Matelica, Italy; sara.sassaroli@unicam.it (S.S.); adolfomaria.tambella@unicam.it (A.M.T.); angela.palumbo@unicam.it (A.P.P.); 3 Clinica Veterinaria San Silvestro, 52043 Castiglion Fiorentino, Italy; f.gallorini@bluvet.it (F.G.); alberto.salvaggio@icloud.com (A.S.); ilariabiagioli27@gmail.com (I.B.)

**Keywords:** bi-oblique proximal ulnar osteotomy, distal ulnar ostectomy, elbow dysplasia, medial coronoid disease, subtrochlear sclerosis

## Abstract

Medial compartment disease is a common occurrence in dogs affected by elbow dysplasia. Despite many treatments suggested in the literature, only few studies reported comparative outcomes in the short and long term. The aim of this study is to report and compare short- and long-term clinical and radiographic outcomes of dogs treated for medial compartment disease (MCD) by distal dynamic ulnar ostectomy (DUO), bi-oblique dynamic proximal ulnar osteotomy (BODPUO) and conservative management (CM). From 2016 to 2018, all medium to large dogs, aged between 5 and 12 months, affected by uni/bilateral MCD and treated by DUO, BODPUO or CM, were enrolled in this study and followed up for 24 months. Orthopedic and radiographic examinations were performed at T_0_, T_2_, T_12_ and T_24_ months after treatment. Lameness score, elbow arthralgia, elbow range of motion (ROM), osteoarthritis (OA) score and percentage of ulnar subtrochlear sclerosis (%STS) were evaluated at each time point. According to the treatment performed, dogs were divided into three groups: DUO, BODPUO and CM. Forty-five elbows from twenty-six dogs, treated with DUO (n = 17), BODPUO (n = 17) or CM (n = 11), were prospectively enrolled in the study. The patients enrolled in the CM group were older and showed more severe radiographic signs of OA, compared to those enrolled in the other two groups. Lameness and arthralgia scores (*p* < 10^−4^) were significantly decreased in patients that underwent surgical treatment and increased in patients managed conservatively (lameness *p* < 10^−4^, arthralgia *p* = 0.3068), at T_12_ and T_24_. OA score (*p* < 0.0040) and ROM (DUO, CM *p* < 10^−4^; BODPUO *p* = 0.0740) worsened in every study group, but %STS decreased in DUO (*p* = 0.0108), increased in the CM group (*p* = 0.0025) and remained unchanged in the BODPUO group (*p* = 0.2740). This study supports the clinical efficacy of DUO and BODPUO in reducing lameness, arthralgia and progression of %STS. Early diagnosis and surgical attention in patients affected by MCD can improve the short- and long-term outcome and reduce the progression of secondary changes.

## 1. Introduction

Elbow dysplasia (ED) is a polygenic and multifactorial disease most commonly affecting young large breed dogs. ED includes medial coronoid process disease (MCPD), osteochondrosis/osteochondritis dissecans (OC/OCD) of the humeral trochlea, ununited anconeal process (UAP) and joint incongruence (INC). These conditions can occur alone, or in association with each other [1,2,3,4,5,6,7,8,9,10,11].

MCPD is the most prevalent condition and encompasses fragmentation (FCP), fissuring, sclerosis and cartilage damage of the medial coronoid process [5,6,7,8,9,10,11]. MCPD can be associated with lesions of the joint cartilage of the humeral trochlea, resulting from humeroulnar conflict (kissing lesions) [12,13,14,15,16,17]. Cartilage degradation and ED primary lesions that affect the medial elbow compartment lead to osteoarthritis (OA) of the medial compartment joint. This advanced stage of ED, involving only the medial aspect of the canine elbow joint, is referred to as medial compartment disease (MCD). Medial compartment disease is characterized by an alternation of inflammation and degeneration of the elbow joint, that compromises dysplastic patients’ quality of life, causing advanced stage of OA, reduced range of motion, pain, lameness and reluctance to move [5,6,7,8,18].

Currently, there is no single treatment for all recognized pathological manifestations, but there are various therapeutic options [5,6,13,16,18,19,20,21,22,23,24,25,26]. The therapeutic choice must be based on the type and extension of the intra-articular lesion, on the severity of pre-existing OA and cartilage damage, on the patient’s age and on the severity of clinical signs [8,27]. 

In recent years, research has been focused on a decision-making algorithm suggesting the most appropriate treatment for the patient [8,27]. Early treatments to correct the suspected underlying cause are preferred in young dogs with absent or minimal articular degeneration. The objective of treatments is to positively impact the development of the disease, slow down the progression of OA and improve patients’ quality of life [8,27]. Early treatments include fragment removal (in case of FCP and OCD), subtotal coronoid ostectomy (SCO) [6], and removal and debridement or replacement of degenerated cartilage [20], in association with dynamic ulnar osteotomy/ectomy that aims to homogeneously distribute the intra-articular loads [8,16,27,28,29].

Distal dynamic ulnar ostectomy (DUO) and bi-oblique dynamic proximal ulnar osteotomy (BODPUO) are early surgical procedures [8,27,30,31] that rely on the forces acting on the proximal ulnar segment to allow it to displace into a more appropriate position, dictated by the action of soft tissues, articular interface interaction and loading forces [9,28,32,33]. The orientation of the proximal ulna relative to the radius and humerus changes when it is unconstrained after osteotomy, which may reduce humero-ulnar conflict [8,16,29,34].

There is a broad agreement about the necessity of an early diagnosis and treatment for a better prognosis [12]. Only few studies directly compared different treatments and a prospective analysis with objective measurements of clinical development and OA evolution in patients treated following the algorithm is lacking. The aim of this article is to report and compare short- and long-term outcomes obtained from clinical and radiographic evaluation after DUO, BODPUO and conservative management (CM) in dogs affected by MCD. We hypothesized that the clinical outcomes would improve after surgery, while the radiographic evaluation would indicate a constant progression of OA, regardless of the performed treatment. 

## 2. Materials and Methods

### 2.1. Animals, Clinical and Radiographic Examinations

Medium to large breed dogs, of less than a year of age, with unilateral or bilateral MCD, were prospectively enrolled in the study. Dogs affected by any other orthopedic or neurologic condition were excluded from the study. Patients underwent complete orthopedic and neurologic examination. Standard International Elbow Working Group (IEWG) radiographic projections of the affected elbows (neutral mediolateral, flexed mediolateral and craniocaudal 15° pronated) were acquired to confirm MCD diagnosis. When the MCD diagnosis was confirmed, the recruited dysplastic joints were divided into study groups according to the therapy received: DUO, BODPUO or CM (DUO, BODPUO and CM groups, respectively).

Clinical and radiographic evaluations were performed by an expert orthopedic surgeon (A.P.P) the day of treatment, or when the CM was started, (T_0_), at 2 months (T_2_), at 12 months (T_12_) and at 24 months (T_24_) after treatment. Age, breed, body weight (BW), body condition score (BCS), lameness and arthralgia scores and ROM were recorded at each time point by the same operator. 

The degree of lameness and arthralgia was assessed by an expert orthopedic surgeon (A.P.P) using a modified Numerical Rating Scale (NRS) proposed by Vasseur et al. (1995) [35,36] (Table 1). ROM was clinically measured with an orthopedic goniometer [37].

**Figure 1:**
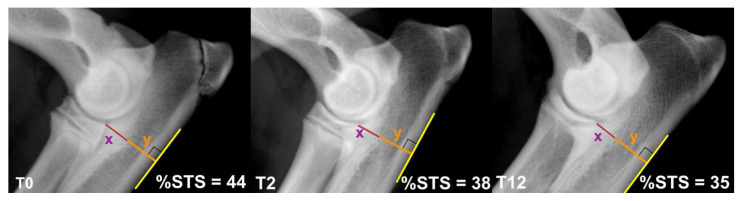
The %STS was calculated as 100(x/y). The craniocaudal ulnar depth (y) was measured from the most proximocaudal aspect of the radial head to the most caudal margin of the ulnar proximal metaphyseal cortex; the depth of sclerosis (x) was measured from the most proximocaudal aspect of the radial head to the STS caudal border. The figure represents the %STS detected on the same elbow at 0, 2 and 12 months from DUO.

OA was assessed with the modified IEWG scoring system using the form by Lang et al. 1998: score 0 = normal (grade 0); score 1 = borderline (grade BL); score 2–4 = mild OA (grade 1); score 5–8 = moderate OA (grade 2); score > 8 severe OA (grade 3) [38]. The same IEWG modified scoring system was used to classify the INC, based on magnitude of radio-ulnar and humero-ulnar steps: mild INC, step < 2 mm; moderate INC, <4 mm; severe INC, >4 mm. The subtrochlear sclerosis of the ulna (STS) was measured using a percentage scale (% STS), as previously described [10,39,40,41] (Figure 1). 

### 2.2. Treatment

The selection of the appropriate treatment for each patient was performed according to the treatment algorithms available in the literature [8,12,27,30]. 

Four- to six-month-old symptomatic puppies with mild radiographic changes (presence of STS without osteophytes and/or mild INC and MCPD) were treated with DUO surgery. Four- to eight-month-old symptomatic dogs with more severe radiographic changes (presence of STS and moderate INC, MCPD and/or OCD-kissing lesion) were treated with BODPUO surgery. 

Four- to twelve-month-old symptomatic puppies with radiographic signs of severe MCD (severe INC, MCPD and/or OCD-kissing lesion and sign of OA) were treated with CM. CM was performed also in dogs where surgical options were declined by the owners. Conservative management consisted of weight control, a joint-type diet, modulation of on-lead exercise and 14 days of oral carprofen (4 mg/kg for 7 days followed by oral carprofen 2 mg/kg for 7 days). Administration of carprofen was repeated as needed.

Patients from DUO and BODPUO groups also underwent a diagnostic elbow arthroscopy. If present, fissure/fracture of the medial coronoid process was arthroscopically removed. A modified Robert Jones bandage was applied for 24 h postoperatively and carprofen (4 mg/kg orally once daily) was administered for 7 days in all dogs. Physical activity was restricted during postoperative phase with lead walks for 2 months. A weight control and joint-type diet were subsequently prescribed. 

### 2.3. Statistical Analysis

Degree of lameness and arthralgia, BCS and OA scores were compared between groups using Kruskal–Wallis test followed by Dunn’s multiple comparison test, or using Mann–Whitney test, where appropriate, at each time point. Friedman analysis followed by Dunn’s test were used to perform a multiple comparison between different time points within each group. 

Cardinal data were assessed for normality using D’Agostino–Pearson test. Range of motion and %STS were compared between groups using One-Way ANOVA analysis followed by Holm–Sidak post hoc test or using Student’s *t*-test, where appropriate. A comparison between different time points within each group was performed using Repeated Measures ANOVA followed by Holm–Sidak test.

Statistical analysis was performed in GraphPad Prism, version 8.2.1 (GraphPad Software Inc., San Diego, CA, USA) and *p* < 0.05 was considered statistically significant.

## 3. Results

### 3.1. Animals, Diagnosis and Treatment

Twenty-six dogs (45 elbows) were enrolled in the study. Thirteen breeds were represented: nine Labrador Retriever, three German Shepherd, two Boxer, two White Swiss Shepherd Dog, two Border Collie, one Saint Bernard, one Chow Chow, one Bernese Mountain Dog, one Golden Retriever, one English Bulldog, one Great Dane, one Tchorny Terrier and one American Staffordshire Terrier, for a total of nineteen males and seven females. Nineteen dogs were bilaterally affected and seven unilaterally. Forty-five elbows were enrolled in the study. Twenty-one right and twenty-four left joints were treated. Mean ± SD age at T_0_ was 6.7 ± 1.3 months. Mean ± SD BW and BCS were 25.2 ± 7.9 kg and 4.6 ± 0.6, at T_0_, 27.8 ± 8.5 kg and 4.6 ± 0.6 at T_2_, 33.2 ± 10.3 kg and 5.5 ± 0.9 at T_12_, and 36.8 ± 9.9 kg and 6.4 ± 1.3 at T_24_, respectively. 

The DUO group included 17 elbows, the BODPUO group included 17 elbows and the CM group included 11 elbows (Table 2). In DUO, BODPUO and CM groups, mean ± SD age at the moment of treatment was 5.9 ± 0.6, 6.6 ± 0.6 and 8.0 ± 1.0 months, respectively. BW and BCS means ± SD were 24.3 ± 6.2 kg and 4.6 ± 0.5, 24.2 ± 6.3 kg and 4.6 ± 0.9, and 28.3 ± 10.9 kg and 4.5 ± 0.5 at T_0_, respectively. They were 26.9 ± 6.7 kg and 4.6 ± 0.5, 26.6 ± 7.1 kg and 4.6 ± 0.9, and 30.9 ± 11.7 kg and 4.6 ± 0.5 at T_2_. At T_12_ they were 32.6 ± 8.0 kg and 5.2 ± 0.8, 31.4 ± 9.9 kg and 5.6 ± 0.9, and 36.2 ± 12.3 kg and 5.7 ± 0.8, while they were 38.9 ± 7.8 kg and 5.6 ± 1.0, 35.9±12.1 kg and 7.0 ± 0.6, and 34.7 ± 6.5 kg and 6.5 ± 2.6 at T_24_. 

All the 45 elbows were evaluated after therapy at T_2_ and T_12_, while only 22 joints (13 dogs) were evaluated at T_24_ follow-up. In particular, seven elbows in the DUO group, seven elbows in the BODPUO group and eight elbows in the CM group were evaluated ad T_24_.

Within the BODPUO group, the radiographic follow-ups showed an excessive proximal ulnar displacement in five elbows [28]. Therefore, the BODPUO group was further divided into the BODPUO-D group, which included five joints with proximal segment dislocated, and the BODPUO-ND group, which included 12 joints with proximal segment not dislocated. 

### 3.2. Clinical Evaluation Findings 

**Figure 2:**
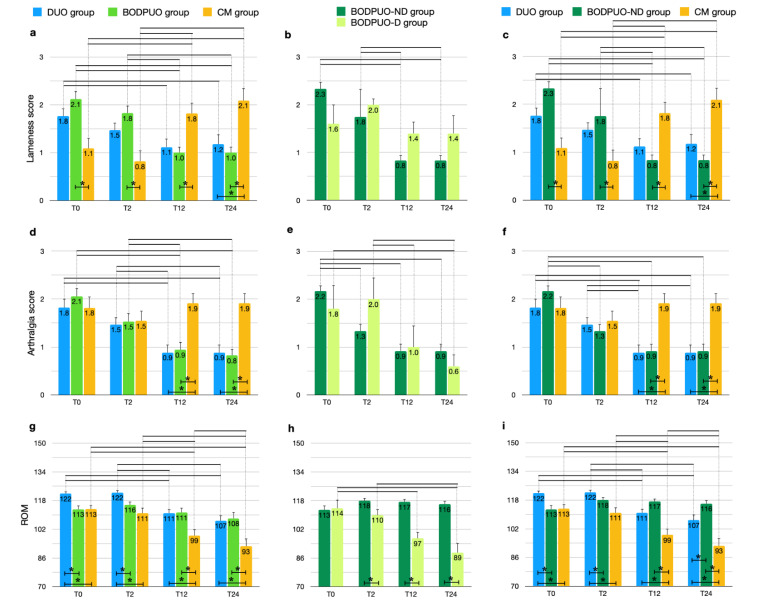
Comparison of lameness score, arthralgia score and ROM (means and SEM) between DUO, BODPUO and CM groups (**a**,**d**,**g**), between BODPUO-ND and BODPUO-D groups (**b**,**e**,**h**), and between DUO, BODPUO-ND and CM groups (**c**,**f**,**i**) at the beginning of treatment (T_0_) and at 2 (T_2_), 12 (T_12_) and 24 (T_24_) months after treatment. Asterisk (*) indicates a significant difference (*p* < 0.05) between groups and the black line (—) indicates a significant difference between time points within the same group.

The lameness score decreased significantly in the DUO group (χ^2^_r_ = 15.00; *p* = 0.0018) and in the BODPUO group (χ^2^_r_ = 31.26; *p* < 0.10^−4^) at long-term evaluation compared with preoperative values (DUO: T_12_ *p* = 0.0065, T_24_ *p* = 0.0140; BODPUO: T_12_, T_24_ *p* = 0.0003). In the CM group the lameness score increased during follow-up (χ^2^_r_ = 25.150; *p* < 10^−4^): at T_0_ and T_2_ it was lower in the CM group compared with BODPUO (T_0_ *p* = 0.0028, T2 *p* = 0.0031) and DUO (T_0_ *p* = 0.1047, T_2_ *p* = 0.0987) groups, but it was higher at T_12_ (DUO *p* = 0.0592, BODPUO *p* = 0.0122) and T_24_ (DUO *p* = 0.0192, BODPUO *p* = 0.0029) (Figure 2a). The results of the comparative statistical analysis performed on the DUO, BODPUO-ND and CM groups (Figure 2c) are very similar to that on the DUO, BODPUO and CM groups. There was no statistically significant difference between the lameness score of patients from the BODPUO and BODPUO-ND groups, when compared to DUO and CM groups (Figure 2c). Lameness score significantly decreased in BODPUO-ND group in long-term evaluations (χ^2^_r_ = 32.44; *p* < 10^−4^) and it was lower than BODPUO-D group at T_2_ (T = 25.50; *p* = 0.6833), T_12_ and T_24_ (T = 15.00; *p* = 0.0924) (Figure 2b) (Table 3).

Arthralgia score decreased in DUO (χ^2^_r_ = 25.47; *p* < 10^−4^) and BODPUO groups (χ^2^_r_ = 31.20; *p* < 10^−4^) at long-term evaluation compared with preoperative values (DUO: T_12_, T_24_ *p* = 0.0009; BODPUO: T_12_, T_24_ *p* < 10^−4^), while in the CM group there was no significant difference between time points (χ^2^_r_ = 3.610; *p* = 0.3068). At T_12_ (H = 12.27; *p* = 0.0022) and T_24_ (H = 14.60; *p* = 0.0007) a higher arthralgia score was detected in the CM group in comparison to DUO (T_12_ *p* = 0.0037, T_24_ *p* = 0.0028) and BODPUO (T_12_ *p* = 0.0072, T_24_ *p* = 0.0013) groups (Figure 2d). In BODPUO-ND the arthralgia scores decreased significantly at T_2_ compared with T_0_ values (*p* = 0.0114). There was no significant difference in arthralgia scores between BODPUO-ND and BODPUO-D groups at each time point (*p* > 0.05) (Figure 2e,f) (Table 3).

ROM significantly decreased in DUO (F = 20.00; *p* < 10^−4^) and in CM (F = 61.88; *p* < 10^−4^) groups at T_24_, while there was no significant difference in BODPUO groups (F = 2.461; *p* = 0.0740). There was significant difference in ROM between groups at each time point. At T_0_ and T_2_ ROM was higher in elbows treated by DUO compared with those treated with BODPUO (T_0_ *p* = 0.0027, T_2_ *p* = 0.0147) and CM (T_0_ *p* = 0.0062, T_2_ *p* = 0.0004) groups. However, at T_12_ and T_24_ DUO (T_12_ *p* = 0.0083, T_24_ *p* = 0.0210) and BODPUO (T_12_ *p* = 0.0083, T_24_ *p* = 0.0167), values were significantly higher compared with the CM group (Figure 2g). ROM significantly decreased in the BODPUO-D group (F = 8.589; *p* = 0.0337), while there was no difference in the BODPUO-ND group (F = 25.48; *p* = 0.1157). ROM in the BODPUO-ND group was higher than in the BODPUO-D group at T_2_ (t = 2.624; *p* = 0.0192), T_12_ (t = 6.472; *p* < 0.10^−4^) and T_24_ (t = 6.295; *p* < 0.10^−4^) (Figure 2h), and significantly higher than in the DUO group at T_24_ (*p* = 0.0378) (Figure 2i) (Table 4). 

There was no significant difference of BCS between all study groups (*p* > 0.05), except for the comparison between BODPUO subgroups (*p* ≤ 0.0123). An improving trend in the BCS was instead appreciated within each group (*p* ≤ 0.0004) (Table 4). 

### 3.3. Radiographic Examination Findings

**Figure 3:**
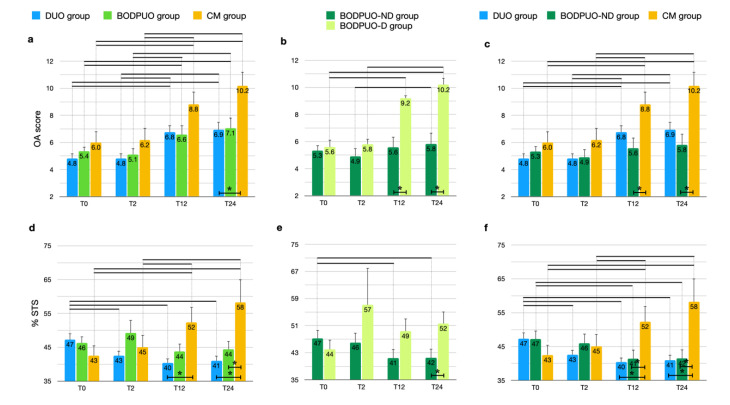
Comparison of OA score and %STS (means and SEM) between DUO, BODPUO and CM groups (**a**,**d**), between BODPUO-ND and BODPUO-D groups (**b**,**e**), and between DUO, BODPUO-ND and CM groups (**c**,**f**) at the time of treatment (T_0_) and at 2 (T_2_), 12 (T_12_) and 24 (T_24_) months after treatment. Asterisk (*) indicates a significant difference (*p* < 0.05) between groups and the black line (—) indicates a significant difference between time points within the same group.

The radiographic OA scores significantly increased in all study groups at long-term evaluations (*p* < 0.05) (Figure 3a). Radiographic signs of OA were more noticeable in the BODPUO-D group than in the BODPUO-ND group at T_12_ (T = 5.000; *p* = 0.0068) and T_24_ (T = 4.500; *p* = 0.0040), because in the BODPUO-D group the score was significantly increased (χ^2^_r_ = 14.47; *p* < 10^−4^) at T_12_ (*p* = 0.0373) and T_24_ (*p* = 0.0022) (Figure 3b). From the comparison of DUO, BODPUO-ND and CM groups there was a difference between BODPUO-ND and CM group at T_12_ (*p* = 0.0577) and T_24_ (*p* = 0.0156). At T_12_ there was no significant difference between BODPUO and CM groups (*p* = 0.3289) (Figure 3c) (Table 5). 

In the BODPUO group the %STS remained almost unchanged during follow-up (F = 1.347; *p* = 0.2740), while in the DUO group it decreased (F = 6.348; *p* = 0.0108) at T_2_ (*p* = 0.0373), T_12_ (*p* = 0.0018) and T_24_ (*p* = 0.0039). In the CM group it increased (F = 13.20; *p* = 0.0025) compared with pretreatment values. The %STS was lower in the DUO group compared to CM group at T_12_ (*p* = 0.0110) and T_24_ (*p* = 0.0050) and it was lower in the BODPUO group compared with the CM group at T_24_ (*p* = 0.0189) (Figure 3d). Comparing the means of %STS in DUO, BODPUO-ND and CM groups, a significant difference was also detected between BODPUO-ND and CM groups at T_12_ (*p* = 0.0235) (Figure 3f). The %STS decreased in the BODPUO-ND group (F = 13.33; *p* = 0.0040) and increased in the BODPUO-D group (F = 0.7874; *p* = 0.4487). At T_24_, the BODPUO-ND group showed significantly lower %STS than the BODPUO-D group (*p* = 0.0481) (Figure 3e).

**Figure 4:**
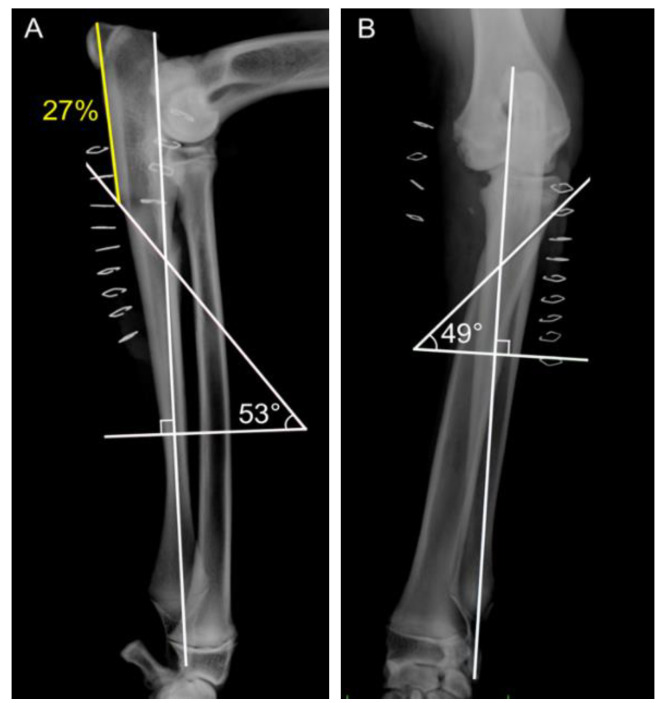
Postoperative medio-lateral (**A**) and cranio-caudal (**B**) radiographic views, following BODPUO, of a patient. The and most caudo-proximal point of osteotomy was situated at 27% of the total ulnar length. (**B**) Latero-medial osteotomy angle was 49°.

In the BODPUO-D group, immediate postoperative radiographs were used to measure the osteotomy geometry. The means ± SD of the caudo-cranial osteotomy angle and the latero-medial osteotomy angles were 53.2 ± 4.9° and 49.7 ± 1.0°, respectively, while the most caudo-proximal point of osteotomy was situated at 32.7 ± 10.0% of the total ulnar length (Figure 4).

## 4. Discussion

This prospective study reports and compares short- and long-term clinical and radiographic outcomes in dogs that underwent surgical and conservative treatment to manage MCD. 

Division into study groups was challenging because of the broad variety of clinical presentations and surgical techniques associated with MCD [5,6,16,18,19,20,21,22,23,24]. This difficulty explains the current paucity of studies that directly compares all techniques proposed by the decision-making algorithm [8,10,19,27,42]. In the present study, the patients were divided according to whether osteotomies were performed and if DUO or BODPUO were executed. 

Ulnar subtrochlear sclerosis is an early sign of elbow dysplasia [30] and has been reported to increase with the progression of the underlying condition [41]. Our results confirmed that the %STS can be useful to assess the progression of the disease, in the short and long term. In addition to that, our results showed that %STS decreased significantly two months after surgery in the DUO group, while it decreased significantly twelve months after surgery in the BODPUO-ND group. This finding supports the hypothesis that DUO and BODPUO may be effective in slowing down the progression of MCD [43]. Our results are consistent with recent clinical studies which showed that the progression of %STS could reduce if the INC is addressed [33,43]. At T_0_, the lameness score in the CM group was lower compared to DUO and BODPUO groups, despite more severe radiographic evidence of OA. However, increased radiographic evidence of OA is not always clinically directly related to the lameness score. It is possible that the increased peri-articular fibrosis associated with the progression of the disease may, to some extent at least in the short term, have increased joint stability and possibly affected the lameness score. In the short term (T_2_), lameness score in the CM group was significantly lower when compared to the DUO and BODPUO groups. This finding can be explained by the expected postoperative recovery time, following surgical treatment, in the DUO and BODPUO groups. However, the clinical long-term outcome obtained in the DUO and BODPUO groups was significantly superior to the CM group. Lameness and arthralgia scores decreased, in the long term (T_12_–T_24_), in patients treated by DUO and BODPUO, according to previous clinical studies [30]. The decreased lameness and arthralgia scores observed in our study might be explained by a homogenous re-distribution of the intra-articular loads, following DUO/BODPUO [29]. 

The ROM decreased and OA score increased in all our study groups. Due to osteophytes and fibrosis interfering with the motion of the joint, moderate inverse correlation between ROM and OA has been previously reported [10,44]. Progression of OA and decreased ROM, in the DUO and BODPUO groups, in spite of an improvement of the clinical outcome, is consistent with previous reviews [10,19,29,45]. 

A recent study demonstrated that BODPUO does not completely restore the INC and increases the humeroulnar rotational instability [29]. The instability could be responsible for the continuous progression of OA and the absence of a clear improvement in ROM. However, at T_12_ and T_24_, the progression of OA and the reduced ROM were significantly lower in the DUO and BODPUO groups compared to the CM.

The ROM of elbows treated by DUO was significantly higher than the mean ROM of BODPUO and CM groups before surgery and at T_2_. This finding was expected, considering that DUO is a surgical procedure recommended in young patients with low cartilage degeneration and with mild clinical symptoms [12,30]. 

Analyzing elbows treated by BODPUO, we suspected that the excessive proximal ulnar displacement in five elbows had a negative impact on the outcome of the BODPUO group. In fact, at long-term evaluation, the results obtained in the BODPUO-D group were significantly worse than the BODPUO-ND group (excluding lameness and arthralgia score, in which there was no statistical difference). 

The mean osteotomy angles and osteotomy position in our study was comparable with what was previously reported by Caron and Fitzpatrick in six elbows with the same complication [28]. In their study, there was no significant difference between the osteotomy angle and position in patients that did and did not develop excessive proximal ulnar displacement. However, the authors supposed that a more acute osteotomy angle or a more proximal osteotomy may lead to excessive motion in some elbows [28]. In a recent study, the authors reported a less severe misalignment than expected if the ulna osteotomy exceeded the recommendations given by Caron and Fitzpatrick [29]. In our study, osteotomies of BODPUO executed too proximally were associated with excessive motion. However, according to recent observations, the excessive motion in our population could also be due to a limited obliquity of the osteotomy [34]. At the long-term follow-up (T_24_), %STS and OA scores were significantly lower in the BODPUO-ND group than in the BODPUO-D group, while the ROM was significantly higher. Surprisingly, in the BODPUO-D group, despite that the lameness score was not improved at T_12_ and T_24_, the arthralgia score was decreased. Considering the low numbers of cases, the subjective assessment of arthralgia, and the theoretical variability of patients’ response to conscious examination, this result is of difficult interpretation. Excluding the BODPUO-D group, the result of the CM, DUO and BODPUO-ND groups, in the present study, are consistent with the data reported in literature [28,30].

This study has several limitations. First of all, the older age and the more severe radiographic changes of the patients in the CM group make the statistical comparison of the data with the DUO and BODPUO group questionable. However, our data support the clinical importance of an early diagnosis and the potential benefits associated with dynamic ulnar osteotomy/ectomy, when case selection is appropriate. On the other hand, the present study highlights how conservative management may have a more limited clinical efficacy in older patients with severe radiographic changes associated with MCD. 

A second limit is the lack of use of CT osteoabsorptiometry to objectively describe bone density distribution in subchondral bone at the level of the base of the medial coronoid process [46]. In this study CT osteoabsorptiometry was not used, but this limitation allowed to stress the utility of evaluating the %STS as an objective parameter that anyone with X-ray equipment can use [47]. 

In the present study, the position of the elbows was standardized, in order to allow repeatable measurements of %STS. However, the INC and the presence of osteophytes on the caudal aspect of the radial head may have affected the assessment of the most proximocaudal aspect of the radial head (a reference point to measure the %STS) [40]. The possibility that %STS may vary depending on the dog breed should be taken into consideration [46]. Therefore, it should be kept in mind that %STS is not a parameter usable to compare individual elbows of dogs of different breeds, but it could be an interesting parameter to assess and monitor the progression of the disease after surgical treatment.

Elbow incongruity was classified by the modified IEWG score during radiographic examination at T_0_, in order to define the severity of radiographic signs and establish the appropriate treatment. Nevertheless, the INC was not assessed in the short and long term, because the radiographic exam did not allow an accurate measurement. In fact, in the past decade, the golden standard for incongruity detection was CT, which provides images without overlapping [48,49].

The absence of a CT scan or arthroscopy did not allow characterization/grading of the severity of the disease in the CM group. The group consisted in older patients with severe radiographic evidence of OA or patients in which the owners declined surgical options. Assessing the outcome of conservative management in such a heterogenous population is difficult.

Moreover, it is widely recognized that keeping the patient’s body condition score at the low end of the normal range slows the progression of degenerative joint disease and the clinical signs associated with it [50]. All the enrolled dogs increased their BCS throughout the study, thereby the weight gain was a conditioning factor and it could be argued that part of the conservative management in the CM group was not adequately performed and that those patients could have had a better clinical outcome if weight control was implemented. 

Several arthroscopic procedures (joint exploration, medial coronoid fragment removal and curettage of the medial compartment) were performed in the DUO and BODPUO groups, as required. The influence of these procedures in the final patients’ outcome is unknown. 

A kinetic and kinematic evaluation of the patients before and after treatment may have objectively confirmed our pre and postoperative subjective evaluation of the lameness. Finally, long-term follow-up was not available for all the patients. 

## 5. Conclusions

This study emphasized the beneficial effects of DUO and BODPUO in reducing lameness, arthralgia and extension of %STS in young patients affected by medial compartment disease. These results highlight the possible capacity of early surgical procedures to homogeneously distribute the intra-articular loads and to slow down and reduce the secondary changes. In particular, this was demonstrated for BODPUO [29], but further research should be conducted regarding the humero-ulnar joint kinematics after DUO surgery. Conservative management in older patients with severe radiographic evidence of elbow OA may be associated with a worse short- and long-term outcome. 

## Figures and Tables

**Table 1:** NRS used to assess the degree of lameness and arthralgia.

Assessment	Grade	Description
Lameness	0	No evidence of lameness neither at the walk nor at the trot
	1	No evidence of lameness at the walk, mild lameness at the trot
	2	Mild lameness at the walk, apparent lameness at the trot
	3	Apparent lameness at the walk and at the trot
	4	No lead of limb during the walk and the trot
Arthralgia	0	No pain response
	1	Head movement, suspension of breath
	2	Subtraction of the limb
	3	Vocalizations, aggressiveness

**Table 2:** Elbows enrolled in the study. Diagnosis and treatment.

Case	Breed	Sex	Age T0 (months)	Weight (kg)	Diagnosis	Group	Associated Therapy
1	German Sheperd	M	6	23	Mild INC, MCPD, STS	DUO	Removal FCP
2	German Sheperd	M	6	23	Mild INC, STS	DUO	None
3	Labrador Retreiver	M	6	24	Mild INC, MCPD, STS	DUO	Removal FCP
4	Labrador Retreiver	M	6	24	Mild INC, MCPD, STS	DUO	Removal FCP
5	German Sheperd	M	5,5	26	Mild INC, MCPD, STS	DUO	Removal FCP
6	Alano	M	6	35	Mild INC, STS	DUO	None
7	Alano	M	6	35	Mild INC, STS	DUO	None
8	White Swiss Sheperd Dog	F	5,5	15	Mild INC, STS	DUO	None
9	Boxer	M	5	18	Mild INC, MCPD, STS	DUO	Removal FCP
10	Boxer	M	5	18	Mild INC, STS	DUO	None
11	Labrador Retreiver	F	5,5	21	Mild INC, MCPD, STS	DUO	Removal FCP
12	Labrador Retreiver	F	5,5	21	Mild INC, MCPD, STS	DUO	Removal FCP
13	Labrador Retreiver	M	6	32,8	Mild INC, MCPD, STS	DUO	None
14	Labrador Retreiver	M	6	32,8	Mild INC, MCPD, STS	DUO	None
15	Labrador Retreiver	F	6	24,5	Mild INC, MCPD, STS	DUO	Removal FCP
16	Labrador Retreiver	M	6	19,8	Mild INC, MCPD, STS	DUO	Curettage medial compartment
17	Labrador Retreiver	M	6	19,8	Mild INC, MCPD, STS	DUO	Curettage medial compartment
18	Labrador Retreiver	M	6,5	22	Moderate INC, MCPD, STS, OCD-kissing lesion	BODPUO	Removal FCP, curettage medial compartment
19	Labrador Retreiver	F	6,5	24	Moderate INC, MCPD, STS	BODPUO	Removal FCP
20	Labrador Retreiver	F	6,5	24	Mild INC, MCPD, STS	BODPUO	Removal FCP
21	Border Collie	F	8	15,5	Mild INC, MCPD, STS	BODPUO	Curettage medial compartment
22	English Bulldog	M	6,5	23,7	Moderate INC, MCPD, STS	BODPUO	None
23	English Bulldog	M	6,5	23,7	Mild INC, STS	BODPUO	None
24	Golden Retreiver	M	5,5	24	Mild INC, MCPD, STS, OCD-kissing lesion	BODPUO	Curettage medial compartment
25	Labrador Retreiver	M	7,5	31	Mild INC, MCPD, STS	BODPUO	Removal FCP, curettage medial compartment
26	German Sheperd	F	6,5	20	Moderate INC, STS	BODPUO	None
27	German Sheperd	F	6,5	20	Moderate INC, STS	BODPUO	None
28	German Sheperd	M	6,5	25,1	Moderate INC, STS	BODPUO	None
29	German Sheperd	M	6,5	25,1	Moderate INC, MCPD, STS	BODPUO	Removal FCP
30	Border Collie	M	7	17	Moderate INC, MCPD, STS, OCD-kissing lesion	BODPUO	Removal FCP, curettage medial compartment
31	Border Collie	M	7	17	Moderate INC, MCPD, STS	BODPUO	Removal FCP
32	Bernese Muntain Dog	M	6,5	40	Moderate INC, MCPD, STS	BODPUO	Removal FCP
33	Saint Bernard	M	6,5	35	Moderate INC, STS	BODPUO	None
34	Golden Retreiver	M	5,5	24	Moderate INC, MCPD, STS, OCD-kissing lesion	BODPUO	Curettage medial compartment
35	White Swiss Sheperd Dog	M	7,5	31,2	Mild INC, MCPD, STS	CM	
36	White Swiss Sheperd Dog	M	7,5	31,2	Mild INC, STS	CM	
37	Amstaff	F	10	20	Moderate INC, MCPD, STS	CM	
38	Amstaff	F	10	20	Moderate INC, MCPD, STS	CM	
39	Chow Chow	F	10	20,5	Severe INC, STS	CM	
40	Chow Chow	F	10	20,5	Severe INC, STS	CM	
41	Labrador Retreiver	M	7,5	31	Mild INC, STS	CM	
42	Tchorny Terrier	M	8	48	Mild INC, STS	CM	
43	Boxer	M	5	20,3	Severe INC, MCPD, STS	CM	
44	Boxer	M	5	20,3	Severe INC, MCPD, STS	CM	
45	Tchorny Terrier	M	8	48	Moderate INC, MCPD, STS, OCD-kissing lesion	CM	

M, male; F, female; INC, joint incongruence; MCPD, medial compartment process disease; STS subtrochlear sclerosis; OCD, osteochondrosis/osteochondritis dissecans; DUO, dynamic ulnar ostectomy; BODPUO, bi-oblique dynamic proximal ulnar osteotomy; CM, conservative management; FCP, fragmentated coronoid process.

**Table 3:** Comparison of lameness and arthralgia (mean ± SD) between study groups and within each group (on the gray rows) at T_0_, T_2_, T_12_ and T_24_.

DUO vs. BODPUO vs. CM									DUO vs. BODPUO-ND vs. CM					DUO vs. BODPUO-ND vs. CM		
		DUO	BODPUO	CM	H	*p*-Value	Post hoc	*p*-Value	BODPUO-ND	H	*p*-Value	Post hoc	*p*-Value	BODPUO-D	T	*p*-Value
**Lameness**	**T0**	1.8 ± 0.7	2.1 ± 0.7	1.1 ± 0.7	10.97	.0042	DUO vs. BODPUO	.5278	2.3 ± 0.5	14.78	.0006	DUO vs. BODPUO-ND	.1110	1.6 ± 0.9	14.00	.1218
							DUO vs. CM	.1047				DUO vs. CM	.1035			
							CM vs. BODPUO	.0028				CM vs. BODPUO-ND	.0004			
	**T2**	1.5 ± 0.6	1.8 ± 0.6	0.8 ± 0.7	10.79	.0045	DUO vs. BODPUO	.5871	1.7 ± 0.4	9.799	.0075	DUO vs. BODPUO-ND	.7959	2.0 ± 1.0	25.50	.6833
							DUO vs. CM	.0987				DUO vs. CM	.0777			
							CM vs. BODPUO	.0031				CM vs. BODPUO-ND	.0064			
	**T12**	1.1 ± 0.7	1.0 ± 0.5	1.8 ± 0.7	8.797	.0123	DUO vs. BODPUO	>.999	0.8 ± 0.4	10.61	.0050	DUO vs. BODPUO-ND	.6885	1.4 ± 0.5	15.00	.0924
							DUO vs. CM	.0592				DUO vs. CM	.0667			
							CM vs. BODPUO	.0122				CM vs. BODPUO-ND	.0041			
	**T24**	1.2 ± 0.8	1.0 ± 0.5	2.0 ± 0.8	11.74	.0028	DUO vs. BODPUO	>.999	0.8 ± 0.4	12.97	.0015	DUO vs. BODPUO-ND	.7304	0.8 ± 0.4	15.00	.0924
							DUO vs. CM	.0192				DUO vs. CM	.0243			
							CM vs. BODPUO	.0029				CM vs. BODPUO-ND	.0014			
	**χ^2^_r_**	15.00	31.26	25.15					32.44					3.000		
	***p*-Value**	.0018	<.0001	<.0001					<.0001					.6667		
**Arthralgia**	**T0**	1.8 ± 0.7	2.0 ± 0.6	1.8 ± 0.7	1.272	.5295	DUO vs. BODPUO	.9513	2.2 ± 0.4	2.543	.2804	DUO vs. BODPUO-ND	.4416	1.8 ± 1.1	22.00	.4289
							DUO vs. CM	>.999				DUO vs. CM	>.999			
							CM vs. BODPUO	>.999				CM vs. BODPUO-ND	.5491			
	**T2**	1.5 ± 0.6	1.5 ± 0.7	1.5 ± 0.7	0.070	.5295	DUO vs. BODPUO	>.999	1.3 ± 0.5	0.545	.7614	DUO vs. BODPUO-ND	>.999	2.0 ± 1.0	18.00	.1370
							DUO vs. CM	>.999				DUO vs. CM	>.999			
							CM vs. BODPUO	>.999				CM vs. BODPUO-ND	>.999			
	**T12**	0.9 ± 0.7	0.9 ± 0.6	1.9 ± 0.7	12.27	.0022	DUO vs. BODPUO	>.999	0.9 ± 0.5	12.95	.0015	DUO vs. BODPUO-ND	>.999	1.0 ± 1.0	28.50	>.999
							DUO vs. CM	.0037				DUO vs. CM	.0026			
							CM vs. BODPUO	.0072				CM vs. BODPUO-ND	.0079			
	**T24**	0.9 ± 0.7	0.8 ± 0.5	1.9 ± 0.7	14.60	.0007	DUO vs. BODPUO	>.999	0.9 ± 0.5	12.95	.0015	DUO vs. BODPUO-ND	>.999	0.6 ± 0.5	21.50	.3458
							DUO vs. CM	.0028				DUO vs. CM	.0026			
							CM vs. BODPUO	.0013				CM vs. BODPUO-ND	.0079			
	**χ^2^_r_**	25.47	31.20	3.610					25.21					10.67		
	***p*-Value**	<.0001	<.0001	.3068					<.0001					.0050		

DUO, dynamic ulnar ostectomy; BODPUO, bi-oblique dynamic proximal ulnar osteotomy; CM, conservative management; H, result of Kruskal–Walls statistics; BODPUO-ND, bi-oblique dynamic proximal ulnar osteotomy with proximal segment not dislocated; BODPUO-D, bi-oblique dynamic proximal ulnar osteotomy with proximal segment dislocated; T0, the day of treatment; T2, two months after treatment; T12, twelve months after treatment; T24, twenty-four months after Table 2. _r_, result of Friedman statistics.

**Table 4:** Comparison of ROM and BCS (mean ± SD) between study groups and within each group (on the gray rows) at T_0_, T_2_, T_12_ and T_24_.

DUO vs. BODPUO vs. CM									DUO vs. BODPUO-ND vs. CM					DUO vs. BODPUO-ND vs. CM		
		DUO	BODPUO	CM	F	*p*-Value	Post hoc	*p*-Value	BODPUO-ND	F	*p*-Value	Post hoc	*p*-Value	BODPUO-D	t	*p*-Value
**ROM**	**T0**	121.9 ± 5.0	113.1 ± 8.6	113.2 ± 7.5	7.880	.0012	DUO vs. BODPUO	.0027	112.8 ± 8.2	8.346	.0010	DUO vs. BODPUO-ND	.0034	113.8 ± 10.5	0.2049	.8404
							DUO vs. CM	.0062				DUO vs. CM	.0042			
							CM vs. BODPUO	.9816				CM vs. BODPUO-ND	.9030			
	**T2**	122.4 ± 5.0	115.6 ± 6.7	110.9 ± 9.7	9.557	.0004	DUO vs. BODPUO	.0147	118.0 ± 5.1	10.01	.0003	DUO vs. BODPUO-ND	.0865	110.0 ± 7.1	2.624	.0192
							DUO vs. CM	.0004				DUO vs. CM	.0002			
							CM vs. BODPUO	.0876				CM vs. BODPUO-ND	.0294			
	**T12**	110.9 ± 9.0	111.3 ± 11.1	98.5 ± 11.2	6.092	.0048	DUO vs. BODPUO	.9083	117.3 ± 5.1	13.53	<.0001	DUO vs. BODPUO-ND	.0614	97.0 ± 7.6	6.472	<.0001
							DUO vs. CM	.0083				DUO vs. CM	.0016			
							CM vs. BODPUO	.0083				CM vs. BODPUO-ND	<.0001			
	**T24**	106.8 ± 12.2	108.1 ± 14.9	92.5 ± 14.1	4.909	.0121	DUO vs. BODPUO	.7848	116.0 ± 6.1	12.36	<.0001	DUO vs. BODPUO-ND	.0378	89.0 ± 11.8	6.295	<.0001
							DUO vs. CM	.0210				DUO vs. CM	.0052			
							CM vs. BODPUO	.0167				CM vs. BODPUO-ND	<.0001			
	**F**	20.00	2.461	25.15					2.548					8.589		
	***p*-Value**	<.0001	.0740	<.0001					.1157					.0337		
**BCS**	**T0**	4.6 ± 0.5	4.6 ± 0.9	4.5 ± 0.5	.0816	.9600	DUO vs. BODPUO	>.999	4.2 ± 0.7	1.656	.4368	DUO vs. BODPUO-ND	.6394	5.4 ± 0.5	7.500	.0123
							DUO vs. CM	>.999				DUO vs. CM	>.999			
							CM vs. BODPUO	>.999				CM vs. BODPUO-ND	>.999			
	**T2**	4.6 ± 0.5	4.6 ± 0.9	4.6 ± 0.5	.0496	.9755	DUO vs. BODPUO	>.999	4.2 ± 0.7	2.145	.3422	DUO vs. BODPUO-ND	.6554	5.4 ± 0.5	7.500	.0123
							DUO vs. CM	>.999				DUO vs. CM	>.999			
							CM vs. BODPUO	>.999				CM vs. BODPUO-ND	.5554			
	**T12**	5.2 ± 0.8	5.6 ± 0.9	5.7 ± 0.8	3.136	.2085	DUO vs. BODPUO	.5548	5.2 ± 0.6	3.863	.1449	DUO vs. BODPUO-ND	>.999	6.6 ± 0.5	3.000	.0016
							DUO vs. CM	.3030				DUO vs. CM	.2192			
							CM vs. BODPUO	>.999				CM vs. BODPUO-ND	.2816			
	**T24**	5.2 ± 0.8	5.9 ± 1.1	6.3 ± 1.4	5.330	.0696	DUO vs. BODPUO	.2562	5.4 ± 1.0	4.595	.1005	DUO vs. BODPUO-ND	>.999	7.0 ± 0.0	5.000	.0010
							DUO vs. CM	.0964				DUO vs. CM	.1039			
							CM vs. BODPUO	>.999				CM vs. BODPUO-ND	.4098			
	**F**	27.00	38.66	30.07					24.19					14.57		
	***p*-Value**	<.0001	<.0001	<.0001					<.0001					.0004		

DUO, dynamic ulnar ostectomy; BODPUO, bi-oblique dynamic proximal ulnar osteotomy; CM, conservative management; F, result of ANOVA F statistics (F-ratio); BODPUO-ND, bi-oblique dynamic proximal ulnar osteotomy with proximal segment not dislocated; BODPUO-D, bi-oblique dynamic proximal ulnar osteotomy with proximal segment dislocated; ROM, range of motion; BCS, body condition score; T0, the day of treatment; T2, two months after treatment; T12, twelve months after treatment; T24, twenty-four months after treatment; X^2^_r_, result of Friedman statistics.

**Table 5:** Comparison of OA and %STS (mean ± SD) between study groups and within each group (on the gray rows) at T0, T2, T12 and T24.

DUO vs. BODPUO vs. CM									DUO vs. BODPUO-ND vs. CM					DUO vs. BODPUO-ND vs. CM		
		DUO	BODPUO	CM	H	*p*-Value	Post hoc	*p*-Value	BODPUO-ND	H	*p*-Value	Post hoc	*p*-Value	BODPUO-D	T	*p*-Value
**OA**	**T0**	4.8 ± 1.4	5.3 ± 1.3	6.0 ± 2.7	2.864	.2388	DUO vs. BODPUO	.5755	5.3 ± 1.3	2.685	.2612	DUO vs. BODPUO-ND	.7042	5.6 ± 1.1	25.00	.6194
							DUO vs. CM	.3676				DUO vs. CM	.3919			
							CM vs. BODPUO	>.999				CM vs. BODPUO-ND	>.999			
	**T2**	4.8 ± 1.4	5.1 ± 1.8	6.2 ± 2.9	2.582	.2750	DUO vs. BODPUO	.7929	4.9 ± 2.1	2.093	.3511	DUO vs. BODPUO-ND	>.999	5.8 ± 0.8	20.50	.3473
							DUO vs. CM	.3775				DUO vs. CM	.4518			
							CM vs. BODPUO	>.999				CM vs. BODPUO-ND	>.999			
	**T12**	6.8 ± 2.1	6.6 ± 2.8	8.8 ± 3.0	3.117	.2104	DUO vs. BODPUO	>.999	5.6 ± 2.6	5.544	.0625	DUO vs. BODPUO-ND	.9119	9.2 ± 0.4	5.000	.0068
							DUO vs. CM	.3530				DUO vs. CM	.3825			
							CM vs. BODPUO	.3289				CM vs. BODPUO-ND	.0577			
	**T24**	6.9 ± 2.3	7.1 ± 3.1	10.2 ± 3.3	7.212	.0272	DUO vs. BODPUO	>.999	5.8 ± 2.7	8.523	.0141	DUO vs. BODPUO-ND	>.999	10.2 ± 1.1	4.500	.0040
							DUO vs. CM	.0382				DUO vs. CM	.0695			
							CM vs. BODPUO	.0627				CM vs. BODPUO-ND	.0156			
	**χ^2^_r_**	44.12	26.69	31.71					13.33					14.47		
	***p*-Value**	<.0001	<.0001	<.0001					.0040					<.0001		
**% STS**	**T0**	47.3 ± 7.2	46.3 ± 7.6	42.5 ± 9.6	1.213	.3077	DUO vs. BODPUO	.7182	47.2 ± 8.2	1.297	.2857	DUO vs. BODPUO-ND	.9843	44.0 ± 6.2	.7881	.4429
							DUO vs. CM	.3595				DUO vs. CM	.3855			
							CM vs. BODPUO	.4154				CM vs. BODPUO-ND	.3855			
	**T2**	42.6 ± 5.3	49.3 ± 15.4	45.1 ± 11.8	1.384	.2620	DUO vs. BODPUO	.2880	46.0 ± 9.7	.5655	.5730	DUO vs. BODPUO-ND	.6844	57.2 ± 24.2	1.402	.1812
							DUO vs. CM	.5902				DUO vs. CM	.7228			
							CM vs. BODPUO	.5902				CM vs. BODPUO-ND	.8083			
	**T12**	40.4 ± 5.0	43.8 ± 9.4	52.4 ± 14.9	4.868	.0127	DUO vs. BODPUO	.3396	41.4 ± 9.1	5.381	.0090	DUO vs. BODPUO-ND	.7969	49.4 ± 8.1	1.692	.1112
							DUO vs. CM	.0110				DUO vs. CM	.0118			
							CM vs. BODPUO	.0592				CM vs. BODPUO-ND	.0235			
	**T24**	41.0 ± 6.0	44.5 ± 9.8	58.3 ± 22.3	6.047	.0050	DUO vs. BODPUO	.4514	41.5 ± 9.2	6.427	.0041	DUO vs. BODPUO-ND	.9226	51.6 ± 7.8	2.152	.0481
							DUO vs. CM	.0050				DUO vs. CM	.0066			
							CM vs. BODPUO	.0189				CM vs. BODPUO-ND	.0096			
	**χ^2^_r_**	6.348	1.347	13.20					3.848					.7864		
	***p*-Value**	.0108	.2740	.0025					.0483					.4487		

DUO, dynamic ulnar ostectomy; BODPUO, bi-oblique dynamic proximal ulnar osteotomy; CM, conservative management; H, result of Kruskal–Walls statistics; BODPUO-ND, bi-oblique dynamic proximal ulnar osteotomy with proximal segment not dislocated; BODPUO-D, bi-oblique dynamic proximal ulnar osteotomy with proximal segment dislocated; OA, osteoarthrosis; %STS, percentage of subtrochlear sclerosis; T0, the day of treatment; T2, two months after treatment; T12, twelve months after treatment; T24, twenty-four months after treatment; X^2^_r_, result of Friedman statistics.

## Data Availability

The data present in this study are available within the article.
